# The Extracellular Vesicle Citrullinome and Signature in a Piglet Model of Neonatal Seizures

**DOI:** 10.3390/ijms241411529

**Published:** 2023-07-16

**Authors:** Subhabrata Mitra, Kelly Harvey-Jones, Igor Kraev, Vinita Verma, Christopher Meehan, Alison Mintoft, Georgina Norris, Ellie Campbell, Katie Tucker, Nicola J. Robertson, Mariya Hristova, Sigrun Lange

**Affiliations:** 1 Department of Neonatology, Institute for Women’s Health, University College London, London WC1E 6BT, UK; k.harvey-jones@ucl.ac.uk (K.H.-J.); v.verma@ucl.ac.uk (V.V.); chris.meehan@ucl.ac.uk (C.M.); a.mintoft@ucl.ac.uk (A.M.); g.norris@ucl.ac.uk (G.N.); ellie.campbell.21@ucl.ac.uk (E.C.); katie.tucker.17@ucl.ac.uk (K.T.); n.robertson@ucl.ac.uk (N.J.R.); 2 Electron Microscopy Suite, Faculty of Science, Technology, Engineering and Mathematics, Open University, Milton Keynes MK7 6AA, UK; igor.kraev@open.ac.uk; 3 Perinatal Brain Repair Group, Department of Neonatology, UCL Institute for Women’s Health, London WC1E 6HU, UK; m.hristova@ucl.ac.uk; 4 Tissue Architecture and Regeneration Research Group, School of Life Sciences, University of Westminster, London W1W 6UW, UK; 5 Pathobiology and Extracellular Vesicle Research Group, School of Life Sciences, University of Westminster, London W1W 6UW, UK

**Keywords:** neonatal, seizure, phenobarbitone, extracellular vesicles (EVs), citrullination/deimination, peptidylarginine deiminase (PAD), liquid biopsy, biomarker, KEGG

## Abstract

Neonatal seizures are commonly associated with acute perinatal brain injury, while understanding regarding the downstream molecular pathways related to seizures remains unclear. Furthermore, effective treatment and reliable biomarkers are still lacking. Post-translational modifications can contribute to changes in protein function, and post-translational citrullination, which is caused by modification of arginine to citrulline via the calcium-mediated activation of the peptidylarginine deiminase (PAD) enzyme family, is being increasingly linked to neurological injury. Extracellular vesicles (EVs) are lipid-bilayer structures released from cells; they can be isolated from most body fluids and act as potential liquid biomarkers for disease conditions and response to treatment. As EVs carry a range of genetic and protein cargo that can be characteristic of pathological processes, the current study assessed modified citrullinated protein cargo in EVs isolated from plasma and CSF in a piglet neonatal seizure model, also following phenobarbitone treatment. Our findings provide novel insights into roles for PAD-mediated changes on EV signatures in neonatal seizures and highlight the potential of plasma- and CSF-EVs to monitor responses to treatment.

## 1. Introduction

Seizures are a common manifestation of brain injury in newborn infants. Controversies still exist over which seizures cause further damage to the developing brain, when to treat them, what drugs to use and how to improve detection. The current evidence base for the management of neonatal seizures is poor, and there is an urgent need for a better understanding of the pathophysiological changes that can guide seizure detection and improved management strategies.

Bicuculline is phthalide isoquinoline. It is a convulsant alkaloid and GABA-receptor antagonist widely used for modeling motor, pain, nociception, anxiety and memory disorders. Bicuculline is often used for the modeling of neonatal seizures in small and large animal models, as it inhibits GABA_A_ receptors on glutamatergic neurons, thus enhancing the excitatory neurotransmitter effects of glutamate [1,2,3,4]. Therefore, bicuculline induces generalized sustained glutamatergic seizures, resulting in increased glutamate release [4]. Bicuculline could be administered either systemically or topically by direct microinjections in selected brain regions [5]. Compared to bicuculline, other GABA_A_ inhibitors (such as picrotoxin, penicillin and pentylenetetrazol), although providing similar induction of seizures, cause side effects [5], which would interfere with readouts observed in humans. Therefore, in our study, we used bicuculline to model neonatal seizures.

Phenobarbitone is the most commonly applied first-line anti-seizure medication used globally in the neonatal population [6]. Previous preclinical data suggest frequent or prolonged seizures at an early stage can be harmful to the developing brain; the most prominent feature in prolonged seizures is cell loss, while altered development is related to briefer recurrent seizures [7]. Hence, it is of pivotal importance to identify molecular pathways that are involved in downstream injury responses and biomarkers that can give indications of response to treatment.

Peptidylarginine deiminases (PADs) cause post-translational modifications of proteins in a calcium-catalyzed manner, by modifying arginine into citrulline. Citrullination/deimination results in changes in protein structure and can affect protein function, and this modification is linked to a range of pathobiological processes [8,9,10,11,12]. Roles for citrullination/deimination have been studied in acute central nervous system (CNS) injury, including neonatal hypoxic-ischaemic brain injury [13,14], spinal cord injury [15] and traumatic brain injury [16,17], as well as in brain cancer [18,19] and neurodegenerative diseases, namely Parkinson’s disease (PD) [20,21,22,23], Alzheimer’s disease (AD) [24,25,26,27], prion disease [28], amyotrophic lateral sclerosis (ALS) [29] and multiple sclerosis (MS) [30,31,32]. Furthermore, a link between citrullination and extracellular vesicle (EV) signatures has been established in several disease models [33,34], including in the CNS [13,19,21,35].

Extracellular vesicles are released from cells as part of cellular communication and carry a range of cargo containing non-coding RNA and proteins, including post-translationally modified proteins. As EVs can be readily isolated from a range of biofluids, they pose as biomarkers for monitoring pathobiological processes and for following responses to treatment. EVs are classified into subpopulations according to size and surface markers, with small EVs (“exosomes” 30–100 nm), medium EVs (“microvesicles” typically 101–350 nm) and large EVs (>300 nm), including apoptotic bodies [36]. The application of EVs as liquid biopsies is increasing in a range of pathologies and in the CNS [37,38,39], but limited research has been carried out in relation to neonatal seizures to date. Citrullination, EV release profiles and their citrullinated protein cargo have been assessed as potential indicators of neuronal damage, including in an in vivo model of PD, showing that the plasma-EV citrullinome mirrors some changes in early PAD-related brain pathology [21].

In this current study, the citrullinomes of both plasma-EVs and cerebrospinal fluid (CSF)-EVs were assessed in a piglet model of neonatal seizures (induced by bicuculine), and in response to a putative treatment with phenobarbitone, the most commonly used anti-seizure medication in neonates, also applied in preclinical piglet brain seizure models [40,41]. The aim was to establish PAD-mediated changes in systemic circulatory EVs (plasma-EVs) and in CSF-EVs, with respect to EV-citrullinome signatures in untreated neonatal seizures and following phenobarbitone treatment to identify whether citrullination may possibly affect critical KEGG pathways. This was assessed using proteomic approaches and protein-interaction network analysis of citrullinated hits of EV cargos from plasma and CSF. In addition, EV numbers and EV size profile changes were investigated.

Our findings provide novel insights into possible roles for PAD-mediated changes in neonatal seizures and highlight the potential of plasma- and CSF-EV signatures to monitor responses to treatment.

## 2. Results

Clear tonic-clonic seizures were noted immediately after bicuculine (BIC) administration, along with concurrent electrographic changes on EEG (Figure S1). The seizure burden was reduced in response to phenobarbitone (PHB). The seizure duration and abnormal seizure duration were scored. BIC treatment significantly increased the seizure duration compared to the control untreated group (*p* = 0.01). Treatment with BIC also significantly increased the duration of abnormal EEG seizures compared to the untreated control group (*p* < 0.0001). Treatment with phenobarbitone reduced the detrimental effect of BIC on the seizure duration, with no significant differences observed in comparison to the control untreated group. However, the reduction in seizure duration was not significant in the PHB + BIC group when compared to BIC alone. PHB reduced the duration of abnormal seizures when compared to BIC alone but with no significant effect, while the duration was still significantly longer when compared to the untreated control groups (*p* = 0.0002) (Figure S1).

### 2.1. Extracellular Vesicle (EV) Characterisation and EV Profiling from Piglet Plasma and CSF

**Figure 1:**
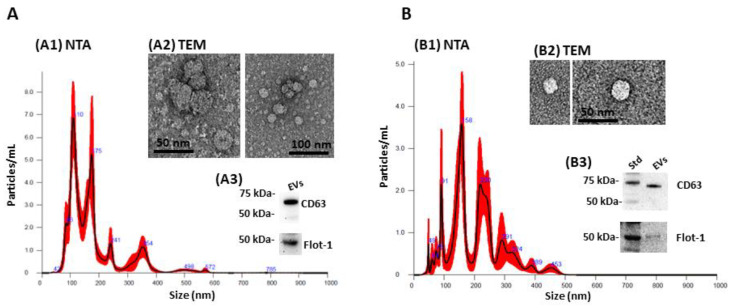
Extracellular vesicle (EV) characterization from piglet plasma and cerebrospinal fluid (CSF). (**A**) Representative plasma-EV profiles are shown by nanoparticle tracking analysis (NTA; **A1**) by transmission electron microscopy (TEM) for morphology (**A2**); and by Western blotting for EV surface markers CD63 and flotillin-1 (**A3**). (**B**) Representative CSF-EV profiles are shown by nanoparticle tracking analysis (NTA); (**B1**) by transmission electron microscopy (TEM) for morphology (**B2**); and by Western blotting for EV surface markers CD63 and flotillin-1 (**B3**). In the NTA graphs (**A1**,**B1**), the black line shows merged data of four 1-min readings per sample and the red line represents the standard error of mean (SEM); for the TEM images scale bars are indicated in nm (**A2**,**B2**) and for the Western blots the protein standard is indicated in kilodaltons (kDa) (**A3**,**B3**).

EV characterization by nanoparticle tracking analysis (NTA) showed that the size distribution of the EVs was in an approximate range of 40–570 nm for plasma-EVs (Figure 1A) and 40–450 nm for CSF-EVs (Figure 1B). EVs were imaged by TEM (Figure 1(A2,B2)) and assessed by Western blotting, showing positive for CD63 and flotillin-1 (Figure 1(A3,B3)).

**Figure 2:**
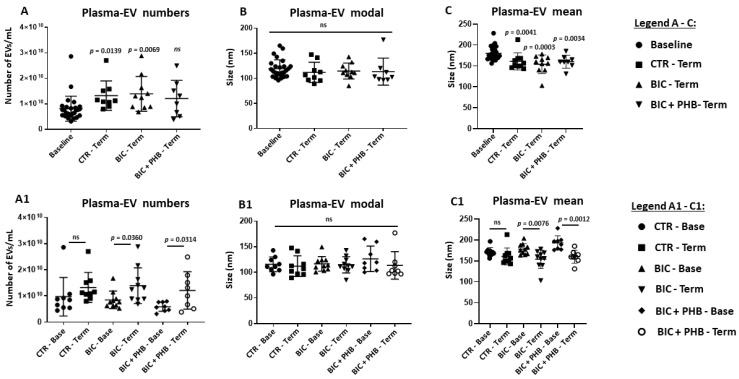
NTA analysis of piglet plasma-EVs from all experimental groups. (**A**) Quantification of plasma-EVs as assessed by NTA analysis, comparing baseline (time zero) to vehicle control group at termination (CTR-Term), seizure group at termination (BIC-Term) and seizure group plus treatment (phenobarbitone) at termination (BIC + PHB-Term). (**A1**) EV numbers in plasma for the same groups but showing each group separately at baseline and terminal points. (**B**) Comparison of modal EV size in plasma, between the four treatment groups. (**B1**) Plasma-EV modal size for the same groups but showing each group separately at baseline and terminal points. (**C**) Comparison of mean EV size in plasma, between the four treatment groups. (**C1**) Plasma-EV mean size for the same groups but showing each group at baseline and terminal point. ANOVA; exact *p*-values are indicated (significant differences at *p* < 0.05; ns = no significant differences. See legend key for individual groups (circles, squares, triangles, diamonds) within the figure.

The quantification of EVs from plasma showed that the total EV numbers were significantly elevated in the terminal groups compared with the zero timepoints at baseline, except for the group that received the phenobarbitone treatment following seizures (BIC + PHB Term) (Figure 2). Here, no statistically significant increase in total plasma-EV numbers was observed compared with the baseline group at time zero (Figure 2A); notably, some outliers were observed for all groups. No significant difference was observed between the groups for the modal size of plasma-EVs (Figure 2B). In addition, the merged mean plasma-EV size was also assessed. Here, a significant decrease in the mean size of plasma-EVs was observed for all the terminal groups (*p* < 0.05) compared with the baseline point (Figure 2C).

To assess intergroup differences (and hence the putative skewing of data due to individual differences), the individual treatment groups were also represented at both base and terminal time points in Figure 2(A1–C1). This showed that the plasma-EV numbers were increased at termination in all groups (albeit not reaching statistical significance for the saline vehicle group (CTR) due to an outlier, while significant differences were observed for the seizure group (BIC) and the seizure group receiving phenobarbitone (BIC + PHB) (Figure 2(A1)). The modal EV size did not differ significantly between groups (Figure 2(B1)), and the mean EV size was significantly reduced at termination for the seizure group (BIC-Term) and the seizure group receiving phenobarbitone treatment (BIC + PHB-Term), while for the saline vehicle group (CTR-Term), no significant change was observed (Figure 2(C1)).

**Figure 3:**
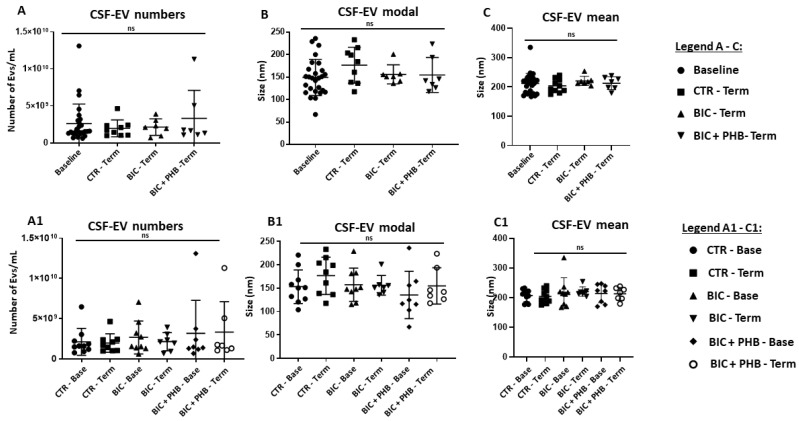
NTA analysis of piglet CSF-EVs from all treatment groups. (**A**) Quantification of CSF-EVs as assessed by NTA analysis, comparing baseline (time zero) to the vehicle control group at termination (CTR-Term), the seizure group at termination (BIC-Term) and the seizure group receiving phenobarbitone treatment at termination (BIC + PHB-Term). (**A1**) EV numbers in CSF for the same groups but showing each group separately at baseline and terminal points; no significant differences between groups (ns). (**B**) Comparison of the modal CSF-EV size between the four treatment groups; no significant differences were observed in modal EV size between the groups. (**B1**) CSF-EV modal size for the same groups but showing each group separately at baseline and terminal points; no significant difference between groups (ns). (**C**) Comparison of the mean EV size in CSF between the four treatment groups; no significant differences were observed in modal EV size between the groups. (**C1**) CSF-EV mean size for the same groups but showing each group at baseline and terminal point; ANOVA; ns = no significant differences. See legend key for individual groups (circles, squares, triangles, diamonds) within the figure.

The quantification of EVs from the CSF showed no significant differences between CSF-EV numbers comparing the terminal groups with the baseline, and in addition, no significant differences were observed between the terminal groups (Figure 3A); some outliers were observed for the base group and terminal group receiving phenobarbitone treatment (BIC + PHB-Term). Comparing the CSF-EV modal size, no significant differences were observed between the four groups (Figure 3B). When assessing the mean EV size in CSF, no significant differences were observed between the groups (Figure 3C), contrary to what was seen in plasma. To assess possible intergroup differences (and hence the putative skewing of data due to individual differences), the different treatment groups were also represented at base and terminal time points for all four groups in Figure 3 (A1–C1), showing that for all groups, CSF-EV numbers, CSF-EV modal size and CSF-EV mean size were not significantly modified.

### 2.2. The Citrullinome of Plasma-EVs and CSF-EVs in a Piglet Model of Neonatal Seizures

**Figure 4:**
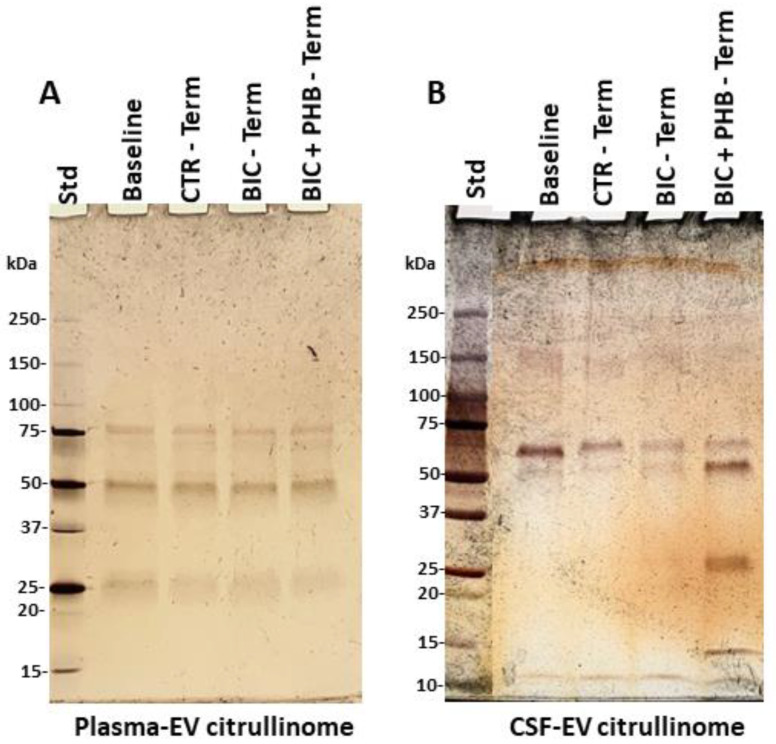
F95-enriched (pan-citrulline) protein fractions are shown by SDS-PAGE and silver staining. (**A**) The citrullinome of plasma-EVs; (**B**) The citrullinome of CSF-EVs. The F95 enriched proteins are shown per lane for the four different groups (Baseline, CTR-Term = saline vehicle control at terminal point; BIC-Term = seizure group at terminal point; BIC + PHB-Term = seizures plus phenobarbitone treatment at terminal point); the protein standard (std) is indicated in the far-left lane in kilodaltons (kDa).

Following plasma-EV isolation, citrullinated proteins were isolated using F95 (pan-citrulline) enrichment in conjunction with LC-MS-MS analysis. This was to identify putative citrullinated protein hits in plasma-EVs of the different treatment groups. The F95-enriched eluted proteins were next assessed by silver-staining following electrophoresis on 4–20% TGX gels, as shown in Figure 4.

The F95 enriched protein eluates were next assessed for the identification of protein hits by LC-MS-MS analysis. Table 1 lists the citrullinated protein hits identified in plasma-EVs in the baseline group, the vehicle control group at termination (CTR-Terminal), the seizure group at termination (BIC-Terminal) and the seizure group receiving phenobarbitone treatment at termination (BIC + PHB-Terminal).

Following CSF-EV isolation, F95 enrichment in conjunction with LC-MS-MS analysis was used to identify putative citrullinated protein hits in CSF-EVs of the different treatment groups. Table 2 lists the citrullinated protein hits identified in the CSF-EVs in the baseline group versus the vehicle control group at termination (CTR-Terminal), the seizure group at termination (BIC-Terminal) and the seizure group receiving phenobarbitone treatment at termination (BIC + PHB-Terminal).

**Figure 5:**
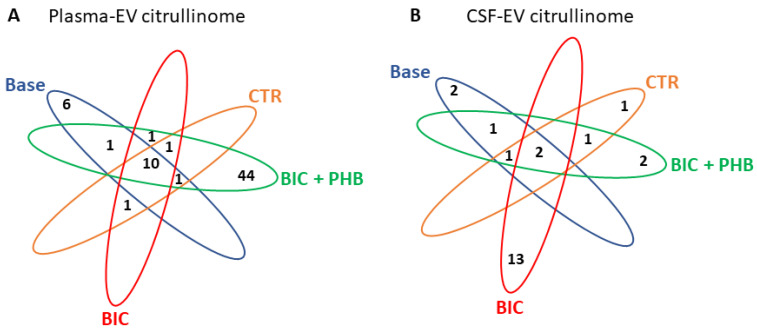
Venn diagrams of the EV citrullinome in a piglet model of neonatal seizure, showing number of citrullinated protein hits in plasma-EVs and CSF-EVs, respectively. (**A**) The number of shared/common citrullinated protein targets and specific ones for individual treatment groups, as identified in plasma-EVs are indicated; (**B**) The number of shared/common citrullinated protein targets and specific ones for individual treatment groups, as identified in CSF-EVs. Baseline samples (Base), vehicle control group at termination (CTR), seizure group at termination (BIC) and the seizure group plus phenobarbitone treatment at termination (BIC + PHB).

The Venn diagrams in Figure 5 represent the number of shared and specific citrullinated protein hits identified in the control and treatment groups in plasma-EVs (Figure 5A) and CSF-EVs (Figure 5B), respectively.

### 2.3. Protein Interaction Network Analysis of the Plasma-EV and CSF-EV Citrullinomes

For protein network analysis, the protein lists for citrullinated candidate hits (from Table 1 and Table 2) were imported into STRING (https://string-db.org/, accessed on 6 January 2023), and protein interaction networks were created for the identification of KEGG (Kyoto Encyclopedia of Genes and Genomes) and GO (Gene Ontology) pathways associated with the citrullinated hits, using medium confidence and the *Sus scrofa* species STRING database (https://string-db.org/, accessed on 6 January 2023).

#### 2.3.1. Protein Interaction Networks for the Plasma-EV Citrullinome

**Figure 6:**
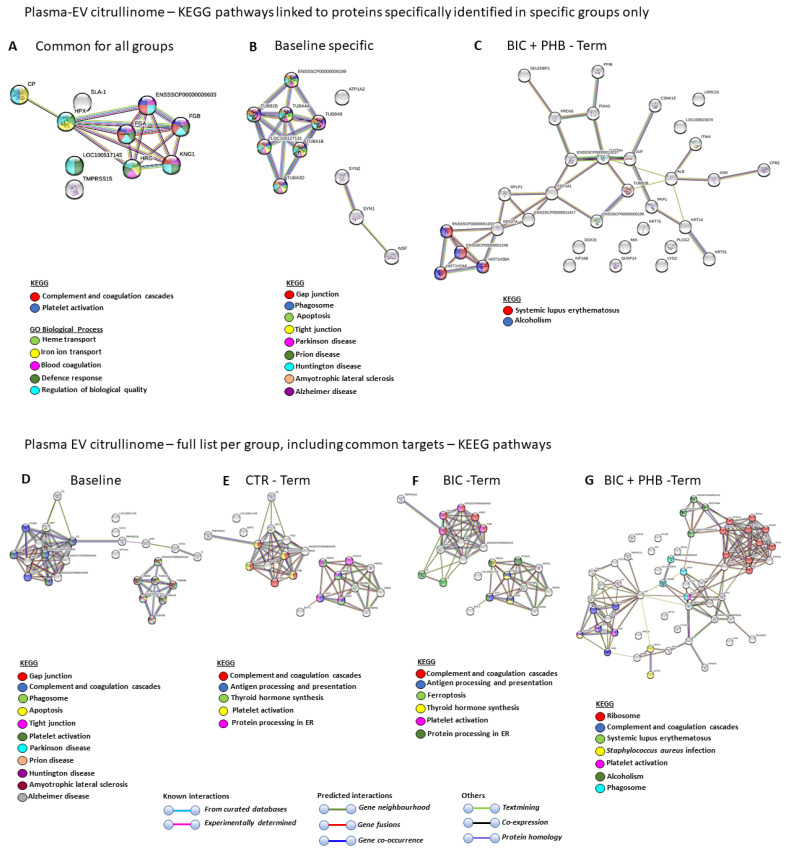
KEEG pathways of the plasma-EV citrullinome. (**A**) KEEG and GO pathways associated with citrullinated protein targets that were common between all treatment groups, including baseline. (**B**) KEGG pathways associated with citrullinated protein targets that were specific for the baseline group only. (**C**) KEGG pathways associated with citrullinated protein targets that were specific only for the seizure group at termination, which received phenobarbitone treatment (BIC + PHB-Term). No KEGG pathways were identified for citrullinated protein targets specific for the baseline terminal group only (CTR-Term) or the seizure terminal point group only (BIC-Term), hence no networks are shown for these. (**D**–**G**): KEGG pathways for the EV citrullinome of each treatment group (Baseline, CTR-Term = vehicle control terminal, BIC-Term =- seizures terminal, BIC + PHB-Term = seizures plus phenobarbitone treatment at terminal point), including common targets, are shown. The different pathways are indicated by the color coding of nodes and associated annotations.

The KEGG pathways associated with the citrullinated protein hits in plasma-EVs that were found to be common between all four groups (including baseline) were complement and coagulation cascades and platelet activation. In addition, the GO biological processes for these common citrullinated protein targets were heme transport, iron ion transport, blood coagulation, defense response and the regulation of biological quality (Figure 6A). The KEGG pathways associated with citrullinated protein hits identified in the baseline group only (and not overlapping with the other groups) were gap junction, phagosome, apoptosis, tight junction, Parkinson’s disease, prion disease, Huntington’s disease, amyotrophic lateral sclerosis and Alzheimer’s disease (Figure 6B). No specific KEGG pathways were identified for targets only specific to the vehicle control terminal (CTR-Term) or seizure terminal (BIC-Term) groups (hence no networks are shown for these), while two KEGG pathways were associated with citrullinated protein targets identified only in the seizure plus phenobarbitone treatment group (BIC + PHB-Term), and these were systemic lupus erythematous and alcoholism (Figure 6C).

When assessing the plasma-EV citrullinome of the full protein hit list per group, including any shared protein hits (therefore hits also common with other groups), eleven KEGG pathways were identified for the baseline group, and these were gap junction, complement and coagulation cascades, phagosome, apoptosis, tight junction, platelet activation, Parkinson’s disease, prion disease, Huntington’s disease, amyotrophic lateral sclerosis and Alzheimer’s disease (Figure 6D). For the plasma-EV citrullinome pathways of the vehicle control terminal group (CTR-terminal), the five KEGG pathways identified were complement and coagulation cascades, antigen processing and presentation, thyroid hormone synthesis, platelet activation and protein processing in endoplasmic reticulum (Figure 6E). For the plasma-EV citrullinome pathways of the seizure terminal group (BIC-terminal), the six KEGG pathways identified were complement and coagulation cascades, antigen processing and presentation, ferroptosis, thyroid hormone synthesis, platelet activation and protein processing in endoplasmic reticulum (Figure 6F). For the plasma-EV citrullinome pathways of the seizure plus phenobarbitone treatment terminal group (BIC + PHB-terminal), the seven KEGG pathways identified were complement and coagulation cascades, systemic lupus erythematosus, *Staphylococcus aureus* infection, platelet activation, alcoholism and phagosome (Figure 6G).

A comparison between the KEGG pathways identified in the four groups for the plasma-EV citrullinome is furthermore summarized in Table 3, highlighting common and unique KEGG pathways associated with the citrullinated protein targets in each treatment group.

#### 2.3.2. Protein Interaction Networks of the CSF-EV Citrullinome

**Figure 7:**
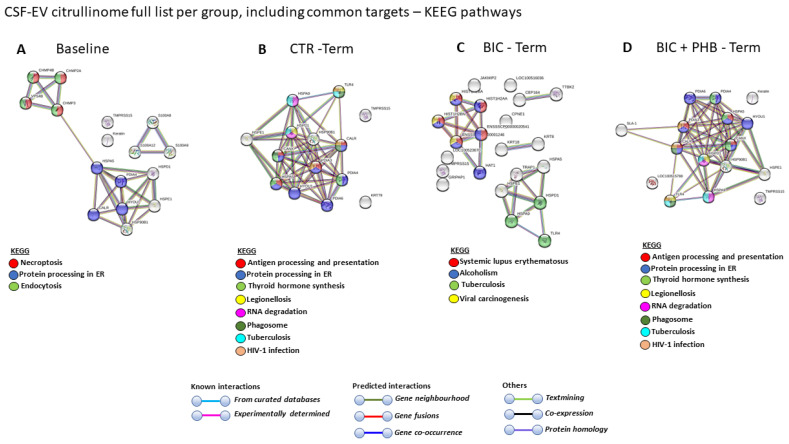
The CSF-EV citrullinome. KEGG pathways associated with citrullinated protein targets identified in each group (including common targets between groups). (**A**) KEEG pathways associated with citrullinated protein targets in the baseline group. (**B**) KEGG pathways associated with citrullinated protein targets in the vehicle control group at termination (CTR-Term) (**C**) KEGG pathways associated with citrullinated protein targets in the seizure group at termination (BIC-Term). (**D**) KEGG pathways associated with citrullinated protein targets in the seizure plus phenobarbitone treatment group at termination (-BIC + PHB-Term). The color coding indicates the different KEGG pathways identified as explained in the accompanying annotations.

The CSF-EV-citrullinome-associated protein interaction networks were analyzed for the four different treatment groups, based on the citrullinated protein targets identified per group and listed in Table 2. For citrullinated hits in CSF-EVs of the baseline group, the following three KEGG pathways were associated: necroptosis, protein processing in the endoplasmic reticulum and endocytosis (Figure 7A). For citrullinated protein hits of the CSF-EVs from the vehicle control terminal group (CTR-Term), the following eight KEGG pathways were associated: antigen processing and presentation, protein processing in the endoplasmic reticulum, thyroid hormone synthesis, legionellosis, RNA degradation, phagosome, tuberculosis and human immunodeficiency virus 1 infection (Figure 7B). For citrullinated protein hits of CSF-EVs in the seizure terminal group (BIC-term), four KEGG pathways were associated: systemic lupus erythematosus, alcoholism, tuberculosis and viral carcinogenesis (Figure 7C). For citrullinated protein hits of CSF-EVs in the seizure plus phenobarbitone treatment terminal group (BIC + PHB-Term), eight KEGG pathways were associated: antigen processing and presentation, protein processing in the ER, thyroid hormone synthesis, legionellosis, RNA degradation, phagosome, tuberculosis and HIV infection (Figure 7D).

A comparison between the KEGG pathways identified in the four groups for the CSF-EV citrullinome is summarized in Table 4, highlighting common and unique KEGG pathways associated with the citrullinated protein targets in each treatment group.

**Figure 8:**
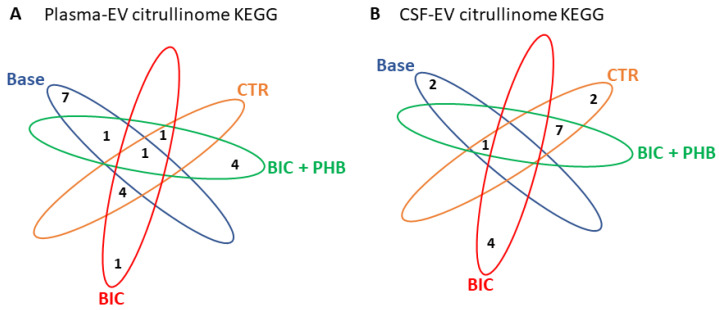
Venn diagrams of KEGG pathways associated with the EV citrullinome in a piglet model of neonatal seizures. (**A**) KEGG pathways associated with the plasma-EV citrullinome; (**B**) KEGG pathways associated with the CSF-EV citrullinome. The number of shared/common KEGG pathways associated with citrullinated target proteins identified for the different treatment groups is indicated. Baseline (Base), vehicle control at termination (CTR), seizure group at termination (BIC), the seizure group + phenobarbitone treatment at termination (BIC + PHB).

The Venn diagrams in Figure 8 represent the number of shared and specific KEGG pathways associated with the citrullinated protein hits identified in the control and treatment groups, in plasma-EVs and CSF-EVs, respectively.

## 3. Discussion

In this study, extracellular vesicle (EV) signatures, including citrullinated protein cargoes, were assessed for the first time as putative biomarkers in neonatal seizures and response to phenobarbitone treatment, using a large animal model of neonatal seizures. When assessing both plasma- and cerebrospinal fluid (CSF)-EVs, more changes were observed in plasma-EV signatures, indicating putative peripheral responses to the insult at an early time point, as replicated in the current model. Plasma-EVs were elevated at termination in all experimental groups, including the control, seizure-induced and phenobarbitone-treated animals, which is possibly indicative of stress responses. Interestingly, the seizure group receiving phenobarbitone did show lower EV numbers closer to the baseline group compared with the untreated seizure group. The modal size of plasma-EVs did not change significantly between the groups, while the mean EV size was significantly reduced in the terminal groups of the seizure (BIC) group and the seizure group treated with phenobarbitone (BIC + PHB). This indicates some change in plasma-EV size profile towards smaller EVs, possibly reflecting a higher level of small EVs released in response to seizures. In comparison, interestingly, CSF-EVs did not show significant changes between the groups or baseline versus termination when assessing the EV numbers, EV modal size or mean EV size. This may indicate that plasma-EVs reflect peripheral changes at the early stages of this specific insult. This may also imply that CSF-EV changes may happen at later time points, which warrants further exploration in future studies.

EVs carry a range of cargo, including RNA, non-coding RNAs and proteins, all of which may be indicative of changes in response to disease and treatment [39,42]. In this study, we focused specifically on post-translationally modified protein cargo with respect to citrullinated/deiminated proteins, due to our ongoing interest in this modification in neuronal injury [8,9,10,13,21,23,35,43,44]. PADs and citrullination, as well as EVs, have previously been linked to CNS injury [8,10,11,13,35] and neurodegeneration [21,23] and may potentially be indicators of modified protein interaction pathways in neonatal seizures. A proteomic analysis was therefore carried out on both plasma-EV and CSF-EV citrullinome cargoes. In plasma-EVs, numerous citrullinated protein candidates were specific for the seizure (BIC) and seizure and phenobarbitone groups respectively (BIC + PHB). Interestingly, for the terminal group of the seizure and phenobarbitone-treated animals (BIC + PHB), this included histones, which are important in gene regulation as well as in extracellular trap formation (Etosis), and their citrullination is indeed linked to such functions, including in the brain [8,45]. Furthermore, histone citrullination has been observed in several CNS injury models, including spinal cord [10] and neonatal hypoxic-ischaemic insult [8], as well as in neurodegenerative diseases including PD [21,23] and in MS [31].

When analyzing the protein-protein networks and associated KEGG pathways linked to the EV citrullinomes in this current study, we found that in plasma-EVs, the following pathways were specific for the seizure group receiving phenobarbitone treatment (BIC + PHB): ribosome, systemic lupus erythematosus, *Staphylococcus aureus* infection and alcoholism. For the untreated seizure group (BIC), ferroptosis was a specific pathway identified for the EV-citrullinome and did not overlap with other groups. The possible relevance of citrullination in these KEGG pathways is briefly discussed below.

The ribosome KEGG pathway was linked to the citrullinome in the seizure group receiving phenobarbitone treatment (BIC + PHB). It has previously been shown that brain protein synthesis is modified and reduced during epileptic seizures (induced by flurothyl or bicuculline) in a 4-day-old rat model [46] and that this may affect brain growth due to neonatal seizures. Interestingly, while deimination is involved in pathological processes, citrullination is also necessary during normal brain development [47]. It may therefore be postulated whether citrullination linked to the ribosome pathway in the phenobarbitone treatment group could be beneficial and allow for repair mechanisms in the CNS, given that citrullination of myelin basic protein also has roles in normal development [48]. Links between citrullination and ribosomal storage in oocytes and in translational control have been established in early mouse embryos [49]. Citrullination is also linked to neuronal stem cell properties, while abhorrent citrullination may lead to neo-epitopes and inflammation-related injury in the CNS [8,30]. Therefore, the role of citrullination in the ribosome pathway may be of interest for further investigation in the current neonatal seizure model.

The systemic lupus erythematosus (SLE) KEGG pathway was linked to the plasma-EV citrullinome in the seizure group receiving phenobarbitone treatment (BIC + PHB). Neonatal lupus erythematosus has previously been linked to CNS disease, including focal seizures [50] and a range of comorbidities, which include seizures [51,52,53]. SLE is an autoimmune disorder and has been linked to citrullination in various studies [6,47,48,54,55], while a link to neonatal seizures has not been previously made in relation to citrullination. Hence, the current findings of modifications in this pathway relating to this post-translational modification via EV-communication may be of some interest.

The *Staphylococcus aureus* KEGG pathway was linked to the plasma-EV citrullinome in the seizure group receiving phenobarbitone treatment (BIC + PHB). This may indicate roles for citrullination in CNS immune responses against bacterial infection, and citrullination is indeed well known to contribute to anti-pathogenic and anti-bacterial functions [56,57]. For example, higher amounts of cyclic citrullinated peptides in sera have been related to infectious diseases, including viral, bacterial and parasitic infections [58], and this KEGG pathway has previously been identified as citrullinated in EV cargo of naked mole-rats under normoxic and hypoxic conditions [59]. The identification of citrullination in this pathway in neonatal seizures here in the current study may possibly be of importance in relation to meningitis and septicemia in neonates [60,61] and seizures in pediatric patients in relation to *S. aureus* infection [62,63,64,65].

The alcoholism KEGG pathway was linked to the plasma-EV citrullinome in the seizure group receiving phenobarbitone treatment (BIC + PHB). This may be relevant for pathways common with alcoholic epilepsy [66], and it may also be considered whether phenobarbitone’s adverse effects [67] may influence citrullination in this pathway. Pre-natal alcohol exposure is linked to increased seizure susceptibility [68,69].

The ferroptosis KEGG pathway was linked to the plasma-EV citrullinome in the seizure group only (BIC). Ferroptosis is an iron-dependent type of cell death triggered by intracellular phospholipid peroxidation, involved in inflammation [70]. Ferroptosis is implicated in pathological cell death in brain injury models, including traumatic brain injury, stroke and neurodegenerative disease [71,72,73]. Interestingly, it has been identified also as one of the pathways associated with neuronal death in neonatal arterial ischemic stroke [74]. Furthermore, the inhibition of the ferroptosis pathway has been identified as a therapeutic target in epilepsy (reviewed in [75]). It may be speculated that ferroptosis, which is classified as regulated necrosis and is considered more immunogenic than apoptosis [70,76], may be involved in inflammatory pathways also associated with the induced seizures in the current model, and this may stimulate citrullination in this KEGG pathway. It is of interest that the ferroptosis pathway was not identified as a citrullinated pathway following phenobarbitone treatment after seizure (BIC + PHB), which may be indicative of a protective function of phenobarbitone in relation to downstream inflammatory responses via citrullination in this pathway, induced by seizures. Investigations into the roles of citrullination with respect to ferroptosis may therefore be of considerable interest.

Interestingly, the only common pathway for the plasma-EV citrullinome in all four groups was the complement and coagulation pathway, linking to complement component C3 being identified as a citrullination candidate in all the groups. The complement system is linked to a range of neurological diseases, including in neonates [77,78,79] and in epilepsy [80,81], and changes in C3 have, for example, been used as an indicator of astrocyte polarisation in response to treatment in a rat epilepsy model [82]. Roles for the complement system are multifaceted in health and disease, with considerable interest in targeting this pathway in various seizure scenarios [83]. Hence, how citrullination may contribute to roles in the regulation of the complement pathway in neonatal seizures and treatment may be of interest and warrants further exploration.

With regards to KEGG pathways associated with the EV citrullinome from the CSF, less marked responses were seen compared with the plasma-EV citrullinome. When analyzing the EVs from the CSF, several citrullinated protein candidates were specific for the seizure groups. This included the following candidates for the untreated seizure group (BIC): IF rod domain-containing protein, histones, kinesin-like protein KIF16B isoform 2, GRIP1 associated protein 1, Janus kinase and microtubule interacting protein 2, tau tubulin kinase 2 and C2 domain-containing protein. In the phenobarbitone treatment group (BIC + PHB), the specific candidates were GLOBIN domain-containing protein and Ig-like domain-containing protein. This links to the various KEGG pathways, although for KEGG pathways relating to the CSF-EV citrullinome, none were specific only to the phenobarbitone treatment group (BIC + PHB). For the seizure group (without treatment; BIC), the following KEGG pathways were specific: systemic lupus erythematosus, alcoholism—both were also found citrullinated in the plasma-EVs of the BIC + PHB group—and tuberculosis and viral carcinogenesis were also specific for plasma-EVs of the seizure group (BIC). This indicates fewer changes with respect to citrullination in response to phenobarbitone treatment in CSF-EVs, compared with plasma-EVs and may reflect differences at this early time point in peripheral versus CSF-mediated response. The tuberculosis and viral carcinogenesis KEGG pathways both link to infection and immune pathways, where citrullination is well known to play significant roles, as discussed above. Tuberculosis can affect the CNS as tuberculous meningitis and relate to pediatric seizures [84]. To what extent citrullination signatures in CSF in this neonatal seizure model are relevant will need further exploration. It may also be considered that the timepoint of assessment in the current study may have resulted in fewer changes in the CSF than in the plasma-EV response, and this will need further investigation in future studies. Another important point to consider is that neonatal seizures could and most often are a consequence of trimester-specific conditions causing maladaptive maternal/placental/fetal triad interactions with the subsequent impairment of neuronal and glial populations as a result of maternal immune activation. Therefore, although in our model, we are able to assess markers affected directly by neonatally caused seizures, we are unable to account for markers altered due to changes in maternal-placental-fetal triad prior to as well as during parturition. Moreover, our model does not provide evidence about secondary detrimental effects from neonatal seizures, given dysautonomia from the primary disease pathway (maternal infection, inflammation, etc.) associated with further brain injury [85,86]. Thus, it is essential that future research and models take into account the gene-environment interactions as a reflection of the continuous process of fetal-neonatal injury before and after birth [85,86].

In this current study, some limitations must be highlighted. This includes the relatively short time frame over which this animal model of neonatal seizures was conducted. This may underestimate the severity spectrum in outcome markers between subjects and could have revealed a better role for PAD-mediated changes if performed at a later time point. Furthermore, this study only used male piglets, limiting the identification of any possible differences in the sex-mediated injury pathway. In models of neonatal CNS injury, sexual dimorphism in behavioral outcome, level of brain damage, mechanisms of cell death and response to treatment have been well documented [87]. Greater memory deficits, especially in tasks requiring spatial memory [88] and caspase-dependent cell death, have been observed predominantly in females, while increased susceptibility to oxidative stress and significant deficit in short-term memory recall [88] have been observed in males.

Importantly, this is the first study to assess EVs as biomarkers in neonatal seizures, opening a platform for further research. EVs have been identified as biomarkers in other neonatal brain injury models, including neonatal stroke [89], acute hypoxic brain injury [90] and in response to therapeutic hypothermia [91]. The potential for EVs as diagnostic biomarkers and for monitoring treatment in neonatal seizures is therefore of considerable interest.

## 4. Materials and Methods

### 4.1. Piglet Model

**Figure 9:**
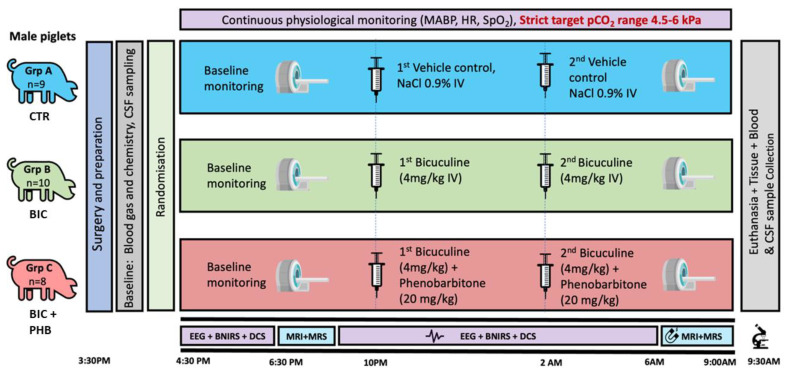
Experimental set-up showing study timeline and details of different groups (A = vehicle control (CTR); n = 9); B = seizure group (Bicuculine (BIC); n = 10); C = seizure plus treatment group (Bicuculine plus Phenobarbitone (BIC + PHB); n = 8). Samples for blood and CSF utilized in this study, were collected at two time points: baseline and termination.

Healthy, Large White male piglets (*Sus scrofa*) (24–75 h) (n = 27) were sedated with midazolam (0.2 mg/kg i.m.) and anesthetized with inhaled isoflurane (1.5–3% *v*/*v*). The piglets were mechanically ventilated through tracheostomy (SLE 2000 infant ventilator, Surrey, UK). An umbilical arterial catheter (Vygon, Surrey, UK) was installed for continuous invasive blood pressure monitoring and blood sampling. An umbilical and a cephalic venous catheter (Vygon, Surrey UK) were installed for fluid infusions. Infusions included fentanyl (3 mcg/kg/h) for analgesia and 10% dextrose (60 mL/kg/d) for maintenance fluid. Piglets were placed prone in a custom-built MRI compatible incubator (LMT Medical Systems, Lübeck, Germany). Temperature was maintained at 38 °C throughout using a Criticool thermal regulation system (Belmont Medical Technology, Wokingham, UK). Piglets were continuously monitored with systemic monitoring (MX750, Philips Healthcare, Farnborough, UK, and 1025T, SA Instruments Inc., London, UK), optical neuro monitoring and continuous video electroencephalogram (EEG) (Nicolet, Natus, WI, USA). After surgical procedures were completed, piglets were divided into three cohort groups to either receive normal saline vehicle (control group A (CTR), n = 9), bicuculine 4 mg/kg IV (pH 4–5) over 10 min (seizure group B (BIC), n = 10) to induce seizures or bicuculine 4 mg/kg IV (pH 4–5) over 10 min followed by phenobarbitone loading dose 20 mg/kg over 20 min, given 1 min after bicuculline infusion completed (treated seizure group C (BIC + PHB), n = 8). All cohort infusions were initiated at 0 h and repeated at 3 h. Prior to infusion, all piglets underwent a 2 h baseline monitoring period, followed by a baseline MRI scan. A second MRI scan was performed at 7 h, after which piglets were euthanized with a pentobarbital overdose (for experimental setup see Figure 9). For EEG, the duration of abnormal EEG activity was assessed by counting the number of minutes of abnormal brain activity on EEG for both dosing periods combined (Figure S1).

All animal experiments were approved by the UCL Ethics Committee and performed under the UK Home Office Guidelines [Animals (Scientific Procedures) Act, 1986] and in compliance with the ARRIVE guidelines (Animal Research: Reporting in Vivo Experiments) for how to REPORT animal experiments.

### 4.2. Plasma and CSF Isolation

Plasma and CSF were collected from all animals at baseline and termination of the study (plasma and CSF were utilized in this current study; in addition, fresh and fixed brain tissue samples were collected for future analysis and are not included in the current study). Plasma was separated from whole blood sampled from umbilical arterial catheter, collected into EDTA tubes and centrifuged at 3000× *g* for 10 min. CSF was directly collected via lumbar puncture at the L3/L4 interspinal space using a Terumo 23 G × 5/8” needle (Terumo, Bagshot, UK). Plasma and CSF aliquots (100 µL) were initially frozen at −20 °C and then stored at −80 °C until further analysis.

### 4.3. Extracellular Vesicle Isolation and Characterisation

Commonly “total EVs”, comprised of small and medium-sized EVs (or 30–1000 nm) are isolated via differential and ultracentrifugation approaches and further characterization of surface markers and morphological analysis by transmission electron microscopy, to meet the minimum requirements of the society of the International Society for Extracellular Vesicles [36]. Here, the EVs were isolated from plasma and CSF according to established protocols [59,92]. Plasma and CSF aliquots (100 µL of each per individual animal) were diluted 1:4 in Dulbecco’s phosphate-buffered saline (DPBS) and centrifuged at 4000× *g* for 20 min to remove apoptotic bodies and then ultracentrifuged at 100,000× *g* for 1 h at 4 °C for isolation of total EVs. The EV-enriched pellet was reconstituted (“washed”) in 500 µL ice-cold DPBS and ultra-centrifuged again at 100,000× *g* for 1 h at 4 °C. Thereafter, the final EV pellet was reconstituted in 100 µL ice-cold DPBS. Isolated EVs were then characterized by nanoparticle tracking analysis (NTA) for number count and size profiling by transmission electron microscopy (TEM) for morphology and by Western blotting for surface markers. EV cargos were then analyzed for citrullinated proteins using F95-enrichment in conjunction with liquid chromatography with tandem mass spectrometry (LC-MS-MS) analysis.

### 4.4. Nanoparticle Tracking Analysis (NTA)

To quantify EVs in the plasma and CSF samples of individual animals from all experimental groups, EVs were isolated as described above. EVs from individual preps were then diluted 1:100 in DPBS (10 µL in 990 µL DPBS) and applied to the NanoSight NS300 (Malvern Panalytical, Malvern, UK) at room temperature, using a syringe pump (flow rate 50), recording videos for 4 × 60 sfor each sample with a blue 488 nm laser, camera level 11 and particle numbers approximately 25–35 per frame. The detection threshold for post-recording analysis was set at level 5. Numbers of samples per group that were assessed for plasma- and CSF-EV count and size profiling by NTA analysis, both at baseline and termination time points, were as follows: A (saline vehicle control; CTR) n = 9; B (seizure; BIC) n = 10; C (seizure plus phenobarbitone treatment; BIC + PHB) n = 8.

### 4.5. Western Blotting

EVs isolated from plasma and EVs were analyzed by Western blotting for CD63 and flotillin-1, which are two key EV-surface markers for smaller EVs (“exosomes”) and medium EVs (“microvesicles”), respectively. EV samples were reconstituted in 2× reducing Laemmli sample buffer and run on 4–20% TGX gels (BioRad, Watford, UK) by SDS-PAGE for 50 min at 165 V, then transferred to nitrocellulose membranes by semi-dry Western blotting for 1 h, followed by blocking in 5% BSA in TBS-T. Incubation with primary antibody (CD63 ab216130, Abcam, Cambridge, UK, diluted 1/1000 in TBS-T and flotillin-1 ab41927, diluted 1/2000 in TBS-T) was carried out overnight at 4 °C on a shaking platform, followed by washing in TBS-T and incubation in secondary HPR-conjugated rabbit Ig antibody (diluted 1/3000 in TBS-T; 170-6515, BioRad) for 1 h at room temperature, and visualization was carried out using enhanced chemical luminescence (ECL; Amersham Biosciences, Buckinghamshire, UK) and the UVP BioDoc-ITTM System (Thermo Fisher Scientific, Dartford, UK).

### 4.6. Transmission Electron Microscopy

In preparation for imaging, EV pellets were resuspended in 100 mM sodium cacodylate buffer (pH 7.4). A drop of EV suspension (~3–5 μL) was placed onto a previously glow-discharged TEM grid with carbon support film. The sample was left to be partially air-dried for ~10 min, followed by placing the grid onto a drop of a fixative solution (2.5% glutaraldehyde in 100 mM sodium cacodylate buffer; pH 7.4) for 1 min at room temperature. The grid was then applied to the surface of three drops of distilled water to wash the sample; excess water was removed using filter paper. Staining of the EVs was carried out for 1 min using 2% aqueous Uranyl Acetate (Sigma-Aldrich, Gillingham, UK); excess stain was removed using filter paper and the grid was left to air dry. EVs were imaged by TEM using a JEOL JEM 1400 transmission electron microscope (JEOL, Tokyo, Japan), which was operated at 80 kV, using a magnification of 30,000× to 60,000×. Digital images were recorded using an AMT XR60 CCD camera (Deben, Bury Saint Edmunds, UK).

### 4.7. Proteomic Analysis of Citrullinated EV Protein-Cargo

To establish an overview of citrullinated protein content and changes in response to treatment in EVs isolated from plasma and CSF, samples from individuals were pooled for the four groups experimental groups. For plasma- or CSF-EVs, respectively, as follows: baseline (n = 27, 20 µL per individual); CTR-terminal = vehicle control at termination (n = 9, 30 µL per individual); BIC-terminal = seizure at termination (n = 10, 30 µL per individual); BIC-PHB-terminal = seizure plus phenobarbitone treatment at termination (n = 8, 30 µL per individual).

Then, a 200 µL pool of plasma-EVs or CSF-EVs, respectively, per group, was applied to Catch-and-Release^®^v2.0 immunoprecipitation mini-columns (17-500 Millipore, Merck, UK), together with the F95 pan-citrullination antibody (MABN328, Merck; [93]) and immunoprecipitation was carried out on a rolling platform at 4 °C overnight. The F95-bound proteins were then eluted with a non-reducing elution buffer according to the manufacturer’s recommendations (Millipore), mixed with a 2× Laemmli sample buffer and then run 0.5 cm into a 12% TGX gel by SDS-PAGE. The whole elute per sample group was cut out as one band for in-gel digestion and downstream LC-MS-MS analysis, carried out by Cambridge Proteomics (Cambridge, UK). Protein lists were analyzed by Mascot against the pig (*Sus scrofa*) database (CCP_*Sus_scrofa Sus_scrofa*_20220926 (329912 sequences; 225,888,489 residues) and a general contamination database (cRAP 20,190,401 (125 sequences; 41,129 residues) for quality control. Cut-off was at >43 for individual ion scores, indicating identity or extensive homology (*p* < 0.05).

### 4.8. Protein Interaction Network Analysis

Proteins identified as citrullinated from the F95 enrichment for the four groups above for plasma-EVs and CSF-EVs, respectively, were assessed for protein-protein networks by STRING analysis (https://string-db.org/; accessed on 6 January 2023). Protein names were inserted under the search form “multiple proteins”, using the *Sus scrofa* database and medium confidence. The resulting protein networks were expanded twice and analyzed for KEEG terms, with pathways represented by colored nodes and the lines representing data mining approaches (known interactions—curated databases, experimentally determined; predicted interactions—gene neighborhood, gene fusion, gene co-occurrence; text mining; co-expression or protein homology).

### 4.9. Statistical Analysis

Histograms were prepared (showing mean and error bars presenting standard deviation; SD). The EV data passed the Shapiro–Wilk, Anderson–Darling, D’Agostino–Pearson and Kolmogorov–Smirnov normality tests using GraphPad Prism 9 and were normally distributed. Therefore, parametric statistical analysis (ANOVA) was carried out using GraphPad Prism 9. The EEG data did not pass the normality tests and therefore were assessed with non-parametric tests (Kruskal-Wallis, with Dunn’s post-hoc). Significant differences were considered as *p* ≤ 0.05. NTA graphs were generated by the NTA software (Version 3, Malvern, UK) with the black line showing merged data of four 1-min readings per sample and the red line representing the standard error of mean (SEM).

## 5. Conclusions

Our findings provide novel insights into roles for PAD-mediated changes in neonatal seizures and highlight the potential of plasma-EVs and CSF-EVs to improve our understanding of the pathophysiological impact, monitor responses to seizures and to treatment, including at early time points. As our findings indicate more marked plasma-EV citrullination, compared with CSF-EV citrullinome, it will be of considerable interest to further explore how different time windows post-injury in a longer study with comparable animals from both sexes are reflected in more marked peripheral responses (plasma-EV signatures) versus changes in local CSF-EV signatures.

## Figures and Tables

**Table 1:** The plasma-EV citrullinome of the piglet neonatal seizure model. A summary of citrullinated protein targets identified in plasma-EVs isolated from baseline plasma samples (n = 27), from the control group (CTR) at termination (n = 9), from the group receiving induced seizure (BIC) at termination (n = 10) and the group receiving phenobarbitone treatment following induction of seizure (BIC + PHB) at termination (n = 8). A tick (V) indicates that the protein hit was present in the respective group. See also Supplementary Tables S1–S4 for full information on the identified protein hits.

Protein Hits	Baseline	CTR Terminal	BIC Terminal	BIC + PHB Terminal
Fibrinogen alpha chain	V	V	V	V
Fibrinogen beta chain	V	V	V	V
Fibrinogen gamma chain	V	V	V	V
Trypsinogen isoform X1	V	V	V	V
Ig-like domain-containing protein	V	V	V	V
Histidine-rich glycoprotein	V	V	V	V
Hemopexin	V	V	V	V
Kininogen 1	V	V	V	V
Complement C3	V	V	V	V
Ceruloplasmin	V	V	V	V
Synapsin-1	V			
Sodium/potassium-transporting ATPase subunit alpha	V			
Tubulin, beta 2B class Iib	V			
Tubulin beta 4B class Ivb	V			
Vesicle-fusing ATPase	V			
Synapsin II	V			
Keratin, type II cuticular Hb5				V
IF rod domain-containing protein				V
Keratin 14				V
Keratin 31				V
Keratin 33A				V
Keratin 34				V
Keratin 40				V
Keratin 75				V
SH3 domain-containing protein				V
Selenium binding protein 1				V
Histone domain-containing protein				V
Histone H2A				V
Histone H2B				V
Histone H4				V
Tubulin beta chain				V
14_3_3 domain-containing protein				V
Leucine rich repeat containing 15				V
Peptidyl-prolyl cis-trans isomerase				V
ADP/ATP translocase				V
Albumin				V
Plakophilin 1				V
Tubulin alpha chain				V
Inter-alpha-trypsin inhibitor heavy chain H4				V
Alpha-2-macroglobulin isoform X1				V
Junction plakoglobin				V
Glyceraldehyde-3-phosphate dehydrogenase				V
Protein kinase domain-containing protein				V
Elongation factor 1-alpha				V
40S ribosomal protein S16				V
Phosphoinositide phospholipase C				V
Urb2 domain-containing protein				V
60S acidic ribosomal protein P1				V
Kinesin family member 16B				V
Phosphoglycerate kinase				V
Carboxypeptidase B2				V
Peroxiredoxin-6				V
Heat shock protein family A (Hsp70) member 8				V
Lysozyme g-like protein				V
Protein disulfide-isomerase				V
Dual specificity phosphatase 14				V
RNA helicase				V
Ubiquitin-40S ribosomal protein S27a				V
Keratin 5	V	V		V
60 kDa heat shock protein, mitochondrial	V	V	V	
Actin, gamma 1	V			V
Endoplasmin		V	V	
Serotransferrin			V	V

**Table 2:** The CSF-EV citrullinome of the piglet neonatal seizure model. A summary of citrullinated protein targets identified in CSF-EVs isolated from baseline CSF samples (n = 27), from the saline vehicle control group (CTR) at termination (n = 8), from the group receiving induced seizure (BIC) at termination (n = 8) and the group receiving phenobarbitone treatment following induction of seizure (BIC + PHB) at termination (n = 7). A tick (V) indicates that the protein hit was present in the respective group. See also Supplementary Tables S5–S8 for full information on the identified protein hits.

Protein Hits	Baseline	CTR Terminal	BIC Terminal	BIC + PHB Terminal
Trypsinogen isoform X1	V	V	V	V
60 kDa heat shock protein, mitochondrial	V	V	V	V
EF-hand domain-containing protein	V			
Charged multivesicular body protein 2a	V			
Keratin 79		V		
Keratin, type II microfibrillar, component 7C			V	
IF rod domain-containing protein			V	
Keratin, type I cuticular Ha3-II			V	
IF rod domain-containing protein			V	
Keratin 18			V	
Histone H2A			V	
Histone H2B			V	
Histone H4			V	
Kinesin-like protein KIF16B isoform 2			V	
GRIP1 associated protein 1			V	
Janus kinase and microtubule interacting protein 2			V	
Tau tubulin kinase 2			V	
C2 domain-containing protein			V	
GLOBIN domain-containing protein				V
Ig-like domain-containing protein				V
Keratin 5	V			V
Endoplasmin	V	V		V
IgG heavy chain		V		V

**Table 3:** KEGG pathways of the plasma-EV citrullinome. A summary of KEGG pathways associated with citrullinated protein targets identified in plasma-EVs isolated from baseline plasma samples, from the control group (CTR) at termination, from the group receiving induced seizure (BIC) at termination and the group receiving phenobarbitone treatment following induction of seizure (BIC + PHB) at termination. A tick (V) indicates that the pathway was identified in the respective group.

KEGG PATHWAYS	Baseline	CTR Terminal	BIC Terminal	BIC + PHB Terminal
Apoptosis	V			
Tight junction	V			
Parkinson’s disease	V			
Prion disease	V			
Huntington’s disease	V			
Amyotrophic lateral sclerosis	V			
Alzheimer disease	V			
Complement and coagulation cascades	V	V	V	V
Gap junction	V	V		
Phagosome	V			V
Platelet activation	V			V
Antigen processing and presentation		V	V	
Thyroid hormone synthesis		V	V	
Platelet activation		V	V	V
Protein processing in ER		V	V	
Ferroptosis			V	
Ribosome				V
Systemic lupus erythematosus				V
*Staphylococcus aureus* infection				V
Alcoholism				V

**Table 4:** KEGG Pathways of the CSF-EV citrullinome. A summary of KEGG pathways associated with citrullinated protein targets identified in CSF EVs isolated from baseline plasma samples, from the control group (CTR) at termination, from the group receiving induced seizure (BIC) at termination and the group receiving treatment following induction of seizure (BIC + PHB) at termination. A tick (V) indicates that the pathway was identified in the respective group.

KEGG PATHWAYS	Baseline	CTR Terminal	BIC Terminal	BIC + PHB Terminal
Necroptosis	V			
Endocytosis	V			
Protein processing in ER	V	V		V
Antigen processing and presentation		V		V
Thyroid hormone synthesis		V		V
Legionellosis		V		V
RNA degradation		V		V
Phagosome		V		V
Tuberculosis		V		V
HIV-1 infection		V		V
Systemic lupus erythematosus			V	
Alcoholism			V	
Tuberculosis			V	
Viral carcinogenesis			V	

## Data Availability

The data supporting the study are enclosed in the manuscript and the Supporting Materials section.

## Supplementary Materials

The following supporting information can be downloaded at: https://www.mdpi.com/article/10.3390/ijms241411529/s1, Figure S1: Histogram showing comparison of seizure duration and EEG scoring between the vehicle control group (CTR), the seizure group (BIC) and the seizure group receiving phenobarbitone (BIC + PHB). The duration of abnormal EEG activity was assessed by counting the number of minutes of abnormal brain activity on EEG for both dosing periods combined (Kruskal-Wallis, with Dunn’s post-hoc; * *p* ≤ 0.05). Table S1: F95 enriched proteins in Plasma-EVs at baseline; Table S2: F95 enriched proteins in Plasma-EVs in the vehicle control group at termination (group A; CTR); Table S3: F95 enriched proteins in Plasma-EVs in seizure group at termination (group B; BIC); Table S4: F95 enriched proteins in Plasma-EVs in seizure group receiving phenobarbital treatment at termination (group C; BIC + PHB); Table S5: F95 enriched proteins in CSF-EVs at baseline; Table S6: F95 enriched proteins in CSF-EVs in the vehicle control group at termination (group A; CTR); Table S7: F95 enriched proteins in CSF-EVs in seizure group at termination (group B; BIC); Table S8: F95 enriched proteins in CSF-EVs in seizure group receiving phenobarbital treatment at termination (group C; BIC + PHB).
