# Supplementary figures and images for: The Extracellular Vesicle Citrullinome and Signature in a Piglet Model of Neonatal Seizures
Source: Int J Mol Sci. 2023 Jul 16;24(14):11529. doi: 10.3390/ijms241411529 (PMC10380774; doi:10.3390/ijms241411529)

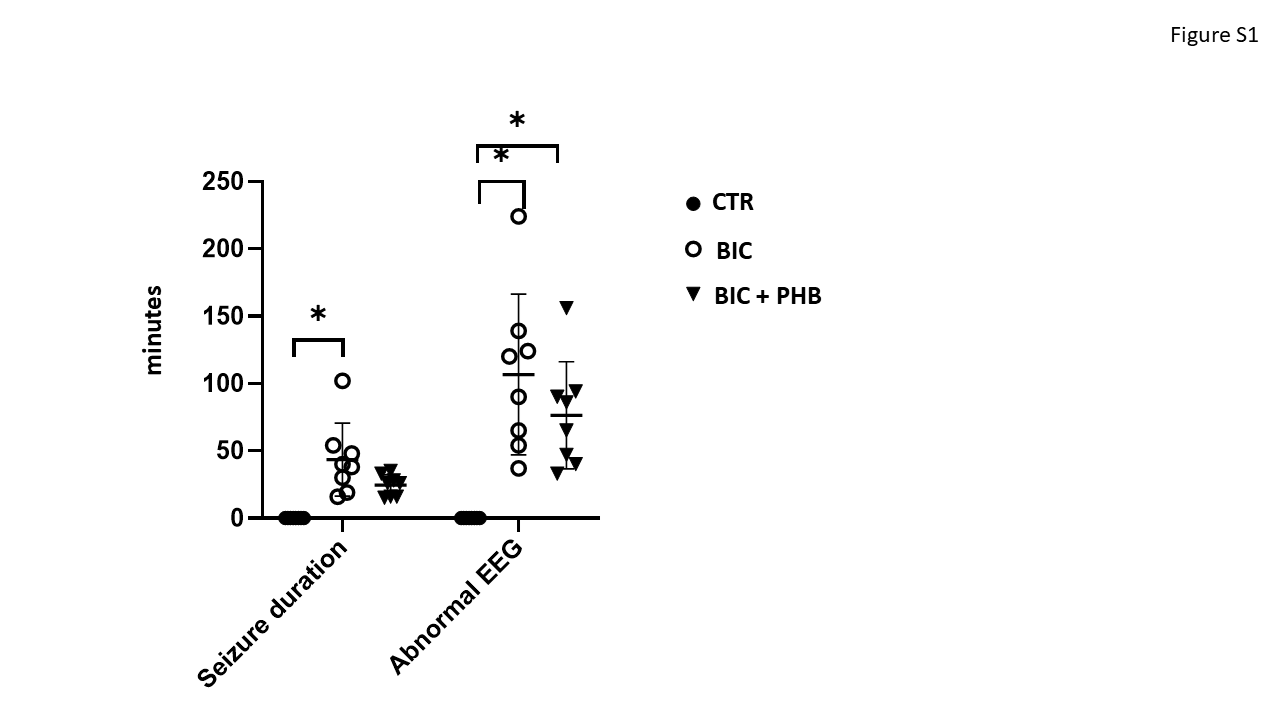

Supplement: Supplementary file 1 [file ijms-24-11529-s001.zip › Fig S1 resubmit.tif]
